# Brassinosteroid-Mediated Resistance to Cobalt-Induced Toxicity by Regulating Hormonal Balance, Cellular Metabolism, and Antioxidant Defense in Maize

**DOI:** 10.3390/plants14132076

**Published:** 2025-07-07

**Authors:** Abdul Salam, Jinzhe Chang, Liupeng Yang, Muhammad Zeeshan, Anas Iqbal, Ali Raza Khan, Muhammad Siddique Afridi, Zaid Ulhassan, Wardah Azhar, Zhixiang Zhang, Peiwen Zhang

**Affiliations:** 1 State Key Laboratory of Green Pesticide, South China Agricultural University, Guangzhou 510642, China; 2 State Key Laboratory for Conservation and Utilization of Subtropical Agro-Bioresources, College of Agriculture, South China Agricultural University, Guangzhou 510642, China; 3 School of Environment and Safety Engineering, Jiangsu University, Zhenjiang 212013, China; 4 Department of Plant Pathology, Federal University of Lavras (UFLA), Lavras 37200-900, MG, Brazil; 5 School of Breeding and Multiplication (Sanya Institute of Breeding and Multiplication), Hainan University, Sanya 572025, China; 6 Zhejiang Key Lab of Crop Germplasm, Department of Agronomy, College of Agriculture and Biotechnology, Zhejiang University, Hangzhou 310058, China

**Keywords:** hormone, brassinosteroids, maize, cobalt stress, cellular metabolism

## Abstract

Brassinosteroids (BRs) play an essential role in regulating plant metabolic pathways that influence growth, development, and stress responses. However, their role in alleviating cobalt (Co) stress has not been extensively studied. This research aimed to assess the impact of exogenous BRs (0.1 µM) on maize subjected to Co stress (300 µM) in a hydroponic experiment. The results indicated that BR supplementation significantly decreased the accumulation of H_2_O_2_ by 17.79 and 16.66%, O_2_^•−^ by 28.5 and 21.48%, and MDA by 37.5 and 37.9% in shoot and root, respectively, as compared to Co stress alone. Additionally, BRs enhanced endogenous levels of BRs (31.16%) and growth hormones (IAA 50.8%, JA 57.8%, GA 52.5%), and reduced Co contents by 26.3% in roots and 36.1% in shoots. BRs enhanced antioxidant enzyme activity both in the shoot and root, leading to reduced ROS levels as confirmed by laser scanning confocal microscopy. Furthermore, BRs increased phenols, flavonoids, and soluble sugars, and elevated total protein content. Observations from transmission electron microscopy indicated reduced ultrastructural damage in plants treated with BRs under Co stress. Taken together, this study highlights the role of BRs in alleviating Co stress in maize, demonstrating their efficiency in enhancing stress tolerance by modulating hormone levels and key metabolic processes.

## 1. Introduction

Plant hormones are chemical messengers produced within plants that regulate a wide range of biological events. They are an essential part of plant growth, development, and stress responses [1]. Brassinosteroids (BRs) are a class of plant hormones belonging to the polyhydroxy steroidal group, primarily involved in various developmental processes such as cell elongation, cell division, and vascular differentiation [2]. Recent studies have shown that BRs exert different mechanisms against HM stress to mediate plant responses and increase stress tolerance. For instance, BRs reduced microplastic stress by improving photosynthesis and redox homeostasis [3], cadmium (Cd) stress by regulating gibberellic acid (GA) accumulation [4] and enhancing nutrient uptake [5], nickel (Ni) stress by reactive oxygen species (ROS) detoxification [6], and chromium (Cr) stress by improving antioxidant defense [7]. BRs modulate key signaling pathways and antioxidant production, which scavenge ROS, enhancing photosynthesis and improving crop yield under stress conditions [8,9]. However, BR signaling is essential for BR-induced tolerance to these stresses, as it regulates downstream gene expression and physiological responses that are critical for stress adaptation [10]. Additionally, BRs establish a dynamic network of crosstalk with other hormones and growth regulators that refine plant growth and stress responses, presenting a viable strategy for agricultural sustainability [11,12].

Soil contamination with excessive heavy metals (HMs) has been a major problem for agricultural production and human health [13,14]. The primary sources of HMs include volcanic eruption, weathering of rocks, and metal corrosion; however, the anthropogenic contribution of HMs to the atmosphere is nearly three times greater than that of natural factors [15]. They are released into soil and water bodies in solution or solid forms, such as synthetic fertilizers, pesticides, sewage sludge, or irrigation with contaminated water, resulting in a high concentration of HMs in soil [16,17]. Unlike organic pollutants, HMs are non-oxidizable and persist in the environment, leading to soil infertility, plant toxicity, and disruption of rhizosphere microbial communities. Cobalt, being a HM, promotes plant growth in trace amounts but becomes toxic in higher concentrations [18]. Previous research has shown that the optimal threshold level of Co in soil or growth media is approximately 50 µM, and any increase beyond this concentration results in impairments to plant cells and membranes, leading to delayed growth and development [19,20]. Reports indicate that one-tenth of China’s woodlands and 60,000 hectares of brownfields in the United States have been identified with HM contamination [21]. Xu et al. [22] reported that the southern parts of China are dominated by tungsten and Co in mangrove sediments. Elevated Co levels in soil induce oxidative stress by generating excessive ROS, which cause ultrastructural damage and disrupt molecular regulation [23]. Co is a redox-active metal that damages plant cells by being directly involved in ROS generation via Haber–Weiss and Fenton-like reactions [24]. However, the uptake and accumulation of Co are largely species-dependent, and certain plant species can survive and grow in soil with higher concentrations, reaching levels between 4000 and 10,000 ppm without showing symptoms of toxicity [17].

Maize (*Zea mays* L.) is a widely cultivated staple food and forage, with annual production reaching approximately 1127 million tons (MT) [25]. Although yields vary by region, maize can grow in a range of agroecological conditions, including diverse temperatures, altitudes, and soil types. North America is the major maize producer, followed by Asia, particularly East Asia. The high yield of maize makes it appealing in densely populated areas with limited land resources [26]. Globally, 61% of maize is used as animal feed, while 13% is consumed directly by humans. It is a key nutritional component for numerous communities in Africa, South Asia, and Latin America, while in East Asia, it mainly serves livestock [27]. However, HM exposure adversely affects plant physiological processes such as photosynthesis, nutrient uptake, and enzyme activity, ultimately impairing overall plant growth and development. Therefore, understanding how plants respond to HM stress is crucial for developing strategies to maintain healthy growth under adverse conditions. Hence, the primary aim of this study is to investigate the impact of BRs on maize under Co stress and to uncover the physiological mechanisms involved. We hypothesize that exogenous BRs may positively regulate plant growth under Co stress by improving hormonal balance, enhancing antioxidant activity, and modulating cellular metabolism.

## 2. Results and Discussions

### 2.1. Effect of BRs on Plant Growth and Co and BR Accumulation

**Figure 1:**
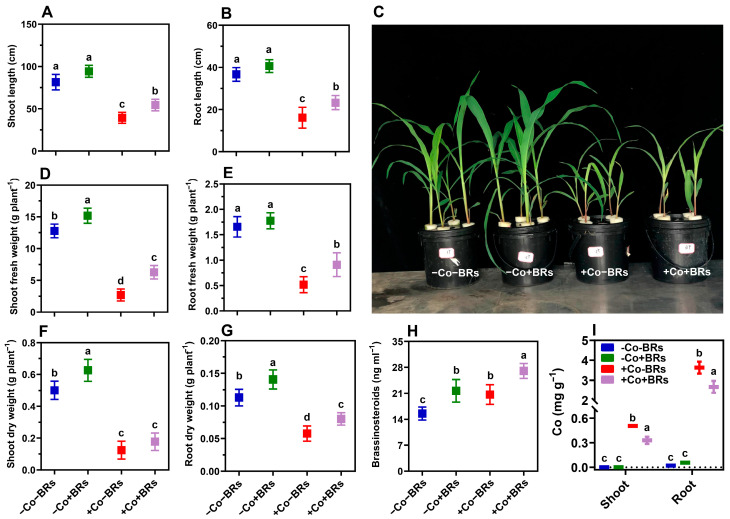
Effects of BRs on maize growth and biomass under Co stress. (**A**) Shoot length, (**B**) root length, (**C**) phenotypic appearance, (**D**) shoot fresh weight, (**E**) root fresh weight, (**F**) shoot dry weight, (**G**) root dry weight, (**H**) brassinosteroid treatment, (**I**) cobalt content in plant tissues. Treatments included control (−Co − BRs), BRs alone (−Co + BRs), Co stress alone (+Co − BRs), and combined BR and Co stress (+Co + BRs). Data represent mean ± SD (n = 3). Different letters indicate significant differences at *p* < 0.05.

Initially, we examined the phenotypic changes in maize plants to investigate how exogenous BRs affect maize’s response to Co toxicity. After 7 days of treatment, seedlings exposed only to Co stress showed significant growth inhibition including stunted growth and chlorosis, compared to the control (Figure 1C). In particular, Co stress reduced shoot length (SL), shoot fresh weight (SFW), shoot dry weight (SDW), root length (RL), root fresh weight (RFW), and root dry weight (RDW) by 51.3, 78.1, 76.1, 55.5, 69.1, and 45.1% as compared to the control. This is because plants exhibit high sensitivity to Co toxicity due to their redox-active nature and the harmful impact of increased Co concentrations [18]. High levels of Co decrease plant biomass and chlorophyll content and alter leaf carbohydrate and phosphorus levels [17]. Additionally, Co stress severely damages root tissues due to their direct exposure, impairing root cell function, reducing nutrient uptake efficiency, and ultimately hindering overall plant growth and development [23,28]. In contrast, exogenous BRs significantly reduced the phytotoxic effect of Co on maize seedlings, improving their phenotypes and morphological attributes. In particular, BRs increased SL, SFW, SDW, RL, RFW, and RDW by 38.3%, 33.2%, 41.1%, 44.32%, 78.3%, and 40.3%, respectively, compared to the Co-treated plants alone (Figure 1A–G). The increase in plant growth and stress tolerance is directly attributed to an elevated level of endogenous BRs. Previously, Piacentini et al. [29] reported that BRs modulate root architecture by enhancing lateral root formation and development in a dose-dependent manner. As key regulators of cell expansion and division, BRs promote diverse plant developmental programs, ranging from cellular processes (division, expansion, elongation) to tissue patterning (vascular development) and stem cell homeostasis [30]. In a study, the knockdown of *ZmBRI1* and its homologs (key receptors in BR signaling) has produced a range of semidwarf maize mutants, underscoring the importance of BR perception in maintaining normal growth [31]. Similarly, *ZmBON1* (a positive regulator of BR signaling in maize) was shown to be essential for BR-mediated growth regulation. Loss-of-function mutations in *ZmBON1* resulted in dwarf phenotypes not due to autoimmunity but rather due to impaired BR signaling [32]. Exogenous BRs promote initial plant growth under Pb stress conditions, with the protective effect attributed to BR-mediated regulation of antioxidant enzymes that reduced Pb toxicity [33]. At the molecular level, BRs confer stress tolerance by modulating central transcription factors, such as BZR1 and BES1, which in turn upregulate downstream genes, including *TCH4* and *CYCD3*. These genes are involved in cell wall remodeling and cell cycle progression, respectively, contributing to enhanced stress adaptation and sustained growth under adverse conditions [34]. BRs have been reported to stimulate cellulose biosynthesis by regulating the expression of *CELLULOSE SYNTHASE* genes, thereby significantly contributing to overall biomass accumulation in *Arabidopsis thaliana* [35].

To further evaluate the effect of BRs on Co accumulation and BR content, we quantified their respective concentrations. The findings demonstrate enhanced accumulation of Co in both the shoot and root tissues, with the maximum accumulation observed in the roots (Figure 1I). Co is regarded as an immobile or relatively less mobile element within plants, resulting in elevated accumulation in root tissues [36]. The higher accumulation of Co in the roots can be attributed to its low mobility, which favors its retention in root tissues, as well as the maize tolerance strategy of restricting its translocation and sequestering it within the roots to limit systemic uptake. Likewise, Tang et al. [34] reported a higher accumulation of Hg in roots compared to shoots, attributing this to the maize tolerance strategy against Hg toxicity, which involves limiting the translocation of Hg to the aerial parts. In addition, BR accumulation was enhanced under Co stress, indicating a possible role in the plant’s adaptive defense mechanisms (Figure 1H). The enhanced accumulation of Co was directly associated with reduced plant growth (Figure 1A–G). However, the application of BRs significantly decreased both Co uptake by 26.3% in roots and its translocation to shoots by 36.1%, thereby limiting systemic accumulation and alleviating toxicity. Sun et al. [4] reported that elevated BR levels in roots modified cell wall hemicellulose composition, reducing Cd uptake in rice. Similarly, the application of BRs in *Brassica napus* significantly decreased Cr accumulation, highlighting the metal-exclusion role of BRs in stress mitigation [37]. These findings are aligned with our results and suggest that BRs contribute to HM stress tolerance by restricting metal uptake, thereby minimizing toxicity and enhancing overall plant tolerance.

### 2.2. Effect of BRs on Light Harvesting and Gas Exchange Attributes

**Figure 2:**
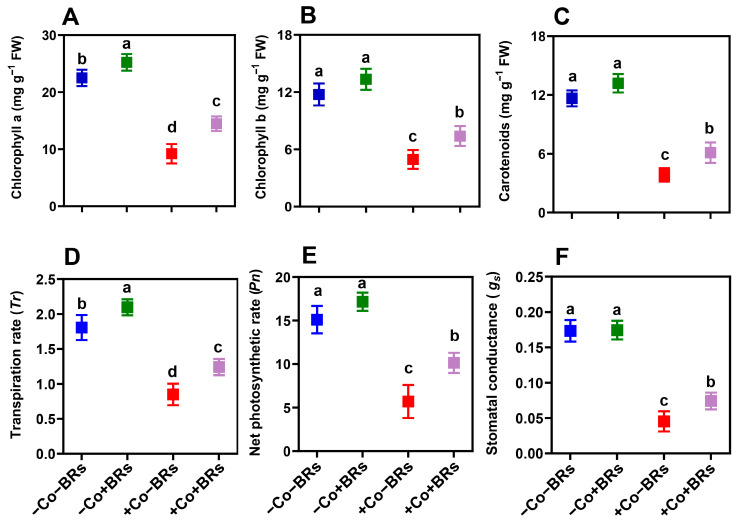
Effects of brassinosteroids (BRs) on photosynthetic pigments and gas exchange parameters under Co stress in maize. (**A**) Chlorophyll a, (**B**) chlorophyll b, (**C**) carotenoids, (**D**) transpiration rate, (**E**) net photosynthetic rate, (**F**) stomatal conductance. Data are presented as mean ± SD (n = 3). Different letters denote statistically significant differences at *p* < 0.05.

We observed an inhibitory effect of Co stress on plant photosynthesis and gas exchange attributes. Specifically, Co stress reduced chlorophyll a, b, and carotenoid contents (Figure 2A–C), alongside significant declines in transpiration rate (*Tr*), net photosynthetic rate (*Pn*), and stomatal conductance (*g_s_*) (Figure 2D–F). These attributes are primarily reduced due to the disruption of photosynthetic pigments (chloroplast ultrastructure), oxidative stress-induced damage (including ROS and membrane damage), and impaired stomatal regulation, collectively hindering gas exchange and overall plant metabolism. Under Co stress, ROS generation in plants disrupts photosystem II (PSII) and impairs the electron transport chain. PSII primary photochemistry is highly sensitive to excess Co^2+^, particularly at higher concentrations [38]. The reduced electron transport beyond the primary quinone acceptor (QA^−^) and the impaired function of the oxygen-evolving complex (OEC) ultimately lead to decreased oxygen production. Additionally, elevated Co levels in the soil reduce photosynthetic pigments and hinder nitrogen metabolism [17]. Heavy metals disrupt photosynthesis by displacing magnesium (Mg^2+^) in chlorophyll, reducing light absorption, and impairing plant photosynthetic function [39].

High concentrations of Co have been reported to adversely affect photosynthesis and gas exchange attributes [18,38]. Contrarily, BRs significantly enhanced light-harvesting pigments (chlorophyll a, b, and carotenoids) and improved gas exchange parameters under Co stress (Figure 2). The increase in these parameters may be due to the ability of BRs to reduce oxidative stress, boost antioxidant enzyme activity, stabilize chlorophyll structure, and enhance photosynthetic efficiency by regulating stomatal conductance and CO_2_ assimilation under HM stress. BRs enhance chlorophyll levels under stress by reducing ROS accumulation, thereby minimizing oxidative damage to the thylakoid membranes and preserving their structural and functional integrity [6]. Xia et al. [40] demonstrated that exogenous BRs enhanced Rubisco activity in cucumber, while brassinazole (a BR biosynthesis inhibitor) suppressed it, suggesting that BRs promote photosynthesis and plant growth by upregulating the synthesis and activation of key photosynthetic enzymes. BRs also stabilize D1 protein to maintain PSII functionality, overcoming stomatal limitations to improve CO_2_ assimilation, and optimizing both light-dependent reactions (electron transport) and dark reactions (Calvin cycle enzymes) for increased carbohydrate synthesis [8]. These findings support our results, indicating that BRs alleviate Co-induced photosynthetic inhibition by safeguarding the photosynthetic machinery, enhancing CO_2_ assimilation, and improving energy efficiency, thereby promoting growth under stress. However, further research is needed to elucidate the precise molecular mechanisms and regulatory networks through which BRs modulate photosynthetic machinery and confer resilience under HM stress.

### 2.3. Effect of BRs on Endogenous Phytohormones

**Figure 3:**
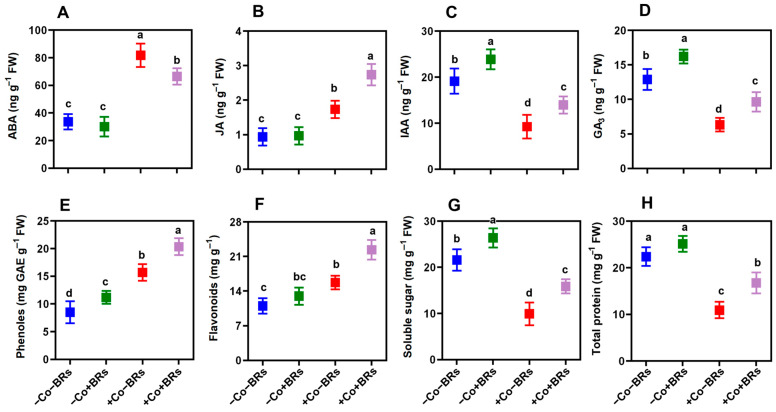
Effect of BRs on key biochemical and hormonal markers in maize exposed to Co stress. Panels illustrate levels of (**A**) Abscisic acid (ABA), (**B**) Jasmonic acid (JA), (**C**) Indole-3-acetic acid (IAA), (**D**) gibberellic acid (GA), (**E**) total phenolics, (**F**) total flavonoids, (**G**) soluble sugars, and (**H**) total protein content. Values represent means ± SD (n = 3). Statistical differences among treatments were determined at significance level of *p* < 0.05. Treatments with different lowercase letters indicate significant differences.

We further quantified major endogenous phytohormones to elucidate the effect of BRs on the hormonal profile. The results demonstrated that BRs exerted a differential regulatory effect on the endogenous hormones. Under non-stress conditions, BRs had no noticeable impact on Abscisic acid (ABA) and Jasmonic acid (JA) (Figure 3A,B). In contrast, significant enhancements were observed in indole acetic acid (IAA) and gibberellic acid (GA_3_) concentrations, which increased by 21.69% and 26.5%, respectively, compared to the control (Figure 3C,D). However, Co stress significantly disrupted hormonal balance, with ABA and JA levels increasing by 143% and 86%, while IAA and GA_3_ levels declined by 51% and 50.9%, respectively, relative to the control (Figure 3A–D). The increase in cellular ABA levels initiates signal transduction and activates genes encoding the plant defense system [41]. This hormonal shift under stress exhibits a transition towards stress-responsive signaling pathways, characterized by increased stress-related and decreased growth-promoting hormone levels, indicating a resource trade-off mechanism between survival and growth [42]. In contrast, the application of BRs under Co stress substantially alleviated these disruptions. BRs reduced ABA levels by 18.74%, indicating an antagonistic interaction, while significantly enhancing the concentrations of IAA, GA_3_, and JA by 50.9%, 52.63%, and 57.8%, respectively, demonstrating synergistic interactions under Co stress (Figure 3A–D). These results emphasize the regulatory role of BRs in modifying hormonal dynamics and maintaining homeostasis under HM stress.

The antagonistic interaction between ABA and BRs has been reported previously, where elevated BR levels suppress ABA biosynthesis, while defects in BR signaling increase plant sensitivity to ABA during key developmental processes, such as root elongation and stomatal closure [43]. The deletion of *BSK5,* a positive regulator of BR signaling, upregulates ABA biosynthesis genes *ABA3* and *NCED3*, supporting this antagonism [44]. Moreover, differential responses to BRs and ABA under cold stress have been observed in maize, where individual treatments alleviated stress but their combination intensified it, impairing plant growth [45]. Similarly, our results showed synergism between BRs and JA, IAA, and GA3, where BRs increased the endogenous level of these hormones and ultimately resulted in Co stress alleviation. Several studies have reported synergistic interactions between BRs and JA during stress adaptation. For instance, BRs enhanced rice seedling tolerance to Cd stress by activating the JA signaling pathway, thereby reducing Cd and ROS accumulation, potentially via the coordinated action of *OsDLT* and *OsMYC2* [46]. Similarly, the combined application of 10 µM JA and 10 µM BR more effectively reduced oxidative stress, as evidenced by decreased ROS, lipoperoxidation, and electron leakage, than individual treatments, indicating a synergistic effect in enhancing stress resilience [47]. BRs also interact positively with GA and IAA signaling. In one study, targeted hormonomics analysis demonstrated that BR application under Cd stress mitigated the reduction in IAA levels and the rise in ACC levels by upregulating the auxin biosynthesis gene *PpYUCCA1* and downregulating the ethylene biosynthesis gene *PpACO1* [42]. This BR-mediated hormonal modulation reduced oxidative stress and enhanced root elongation and lateral root formation, underscoring the crosstalk between BRs, IAA, and ethylene in conferring Cd tolerance in Kentucky bluegrass. Similarly, BRs activate BZR1, which promotes GA biosynthesis by upregulating *GA20ox* genes [48]. GA, in turn, facilitates the degradation of DELLA proteins, which otherwise inhibit BZR1 activity [49]. This reciprocal regulation enhances the expression of BZR1 target genes, promoting cell elongation and seedling development. Under HM stress, the interaction between BZR1 and DELLA also contributes to increased levels of ROS-scavenging enzymes, supporting stress tolerance [50]. Collectively, these interactions demonstrate the integrative role of BRs in regulating hormonal signaling networks to optimize growth–defense trade-offs under abiotic stress conditions.

### 2.4. Effect of BRs on Polyphenol, Total Protein, and Soluble Sugar

In comparison to non-stressed control treatments, BR and Co stress alone resulted in significant increases in phenolic content (31.2% and 84.4%, respectively) and total flavonoid content (18% and 45.3%, respectively) (Figure 3E,F). Plant exposure to Co stress induces the accumulation of phenolic compounds and flavonoids as part of an adaptive defense response to mitigate oxidative damage [23]. Phenolic compounds, known for their antioxidant properties, play a crucial role in scavenging free radicals, as their biosynthesis is upregulated in response to abiotic stress conditions [51]. Similarly, flavonoids stored in vacuoles play a crucial role in *in vivo* ROS scavenging across multiple cellular compartments [52]. Interestingly, BR application under Co stress further increased phenol and flavonoids by 29.73 and 42.32%, respectively, compared to the Co stress alone. The BR-induced increase in phenol and flavonoid contents can be attributed to their role in activating phenylpropanoid pathway enzymes, enhancing secondary metabolite biosynthesis, and augmenting the plant defense system under stress conditions. Phenolic compounds activate the phenylpropanoid pathway, leading to the synthesis of phenylpropanoid derivatives, including phenols and flavonoids, which help alleviate oxidative stress in plants [53]. Previously, Liu et al. [54] reported that exogenous BRs enhanced plant tolerance to zinc stress primarily by modulating the phenylpropanoid biosynthetic pathway, thereby promoting the accumulation of protective secondary metabolites. Similarly, Zhang et al. [55] reported that exogenous BRs significantly increased phenylalanine ammonia-lyase (PAL) activity and upregulated PAL-related gene expression, indicating a positive role of BRs in activating the phenylpropanoid biosynthesis pathway. Additionally, we observed a sharp decline in soluble sugar (62.4%) and total protein contents (56.5%) upon exposure to Co stress (Figure 3G,H). This indicates disruption in primary metabolism and reduced biosynthetic activity, likely due to oxidative damage and impaired physiological functions. In contrast, BR application markedly alleviated these reductions, improving soluble sugar and protein contents by 59.6% and 54.12%, respectively, compared to Co treatment alone. These changes suggest that BRs enhance carbon and nitrogen metabolism to support energy availability and protein synthesis under Co-induced stress. Previously, Sun et al. [4] reported that BRs increased the total sugar content in root cell wall hemicellulose, reduced Cd stress in rice, and enhanced plant growth and stress tolerance. Similarly, Niu et al. [56] demonstrated that BRs enhanced soluble sugar and total protein levels, contributing to improved heat stress tolerance.

### 2.5. Effect of BRs on Oxidative Stress Markers and Antioxidant Defense Enzymes

**Figure 4:**
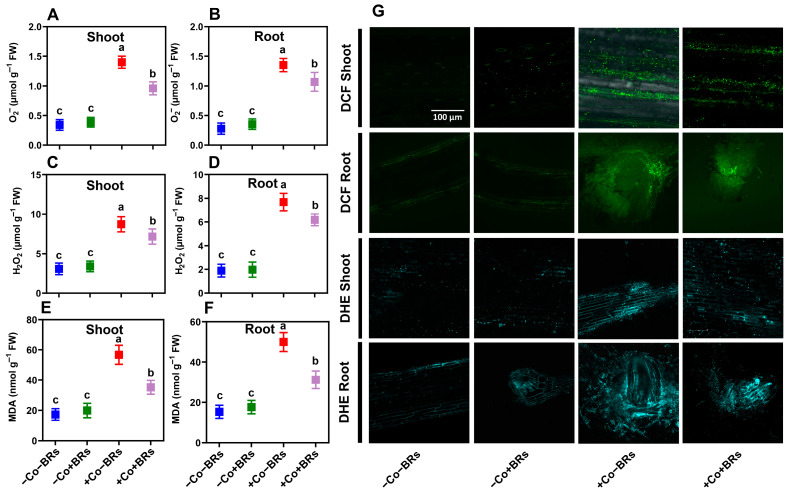
Brassinosteroid-mediated attenuation of oxidative stress markers in maize under Co stress. Quantification of superoxide anion (O_2_^•−^) in shoot (**A**) and root (**B**), hydrogen peroxide (H_2_O_2_) in shoot (**C**) and root (**D**), malondialdehyde (MDA) in shoot (**E**) and root (**F**). Scale bar = 100 µm. Confocal laser scanning microscopy images showing in situ detection of ROS accumulation: DCFH-DA fluorescence for H_2_O_2_ and DHE fluorescence for O_2_^•−^ in both shoot and root sections (**G**). Data are presented as mean ± SD (n = 3), with statistically significant differences at *p* < 0.05. Different lowercase letters indicate significant differences.

**Figure 5:**
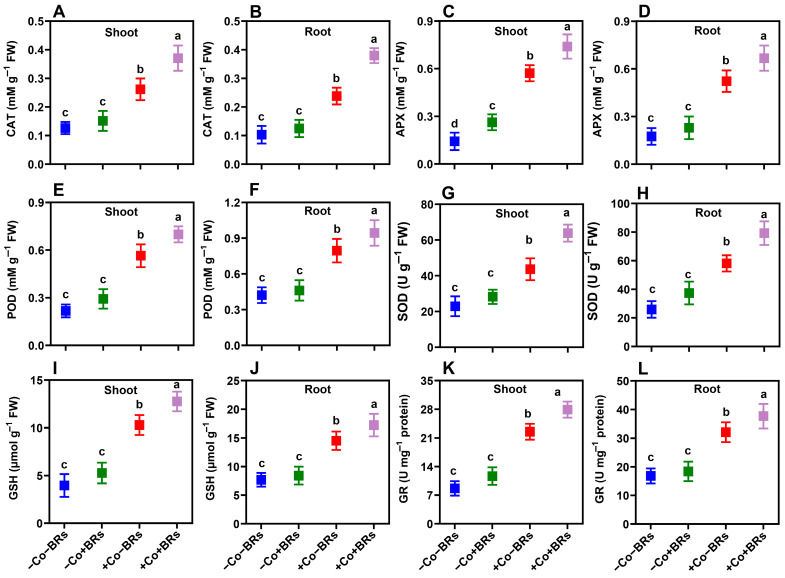
Brassinosteroid-induced enhancement of enzymatic and non-enzymatic antioxidant defense in maize under Co stress. Activities of catalase (**A**,**B**), ascorbate peroxidase (**C**,**D**), peroxidase (**E**,**F**), superoxide dismutase (**G**,**H**), reduced glutathione (**I**,**J**), and glutathione reductase (**K**,**L**) in shoot and root tissues, respectively. BR treatment significantly elevated antioxidant enzyme levels in Co-stressed plants, contributing to ROS detoxification and improved stress tolerance. Data are presented as mean ± SD (n = 3), with statistically significant differences at *p* < 0.05. Different lowercase letters indicate significant differences.

To determine the potential impacts of exogenous BRs on oxidative stress markers and antioxidant enzymes, we initially quantified the concentrations of superoxide anion (O_2_^•−^), hydrogen peroxide (H_2_O_2_), and malondialdehyde (MDA) contents in both leaves and roots of the studied plants (Figure 4). The results indicated no significant difference in these parameters between the control and BR-treated plants, while Co stress notably increased them. Compared to the control plants, Co stress raised H_2_O_2_ levels by 184.3% (shoot) and 306.3% (root), O_2_^•−^ by 308.8% (shoot) and 382.14% (root), and MDA by 226.4% (shoot) and 208.7% (root). Additionally, ROS accumulation was visualized microscopically using H_2_O_2_- and O_2_^•-^-specific probes (DCF and DHE, respectively). The results demonstrated strong agreement with quantitative measurements, showing intense fluorescence signals under Co treatments (Figure 4G). ROS is a natural by-product of cellular metabolism, and its levels increase under conditions that disrupt redox homeostasis, such as exposure to elevated concentrations or prolonged durations of HM stress [57]. The generation of these highly reactive species induces oxidative damage, characterized by lipid peroxidation, cellular membrane denaturation, biomolecular degradation, and DNA strand breakage [58]. Previously, Ali et al. [59] reported elevated levels of H_2_O_2_, O_2_^•−^, and MDA under Co stress and demonstrated that these oxidative markers correlate strongly with visible symptoms of phytotoxicity and growth inhibition. To counteract oxidative stress, plants employ a complex antioxidant defense system that includes both enzymatic and non-enzymatic components, collectively maintaining cellular redox homeostasis [60]. In this study, we observed an increased level of catalase (CAT), ascorbate peroxidase (APX), peroxidase (POD), and superoxide dismutase (SOD) and reduced glutathione (GSH) and glutathione reductase (GR) in both leaves and roots of the studied plants when subjected to Co stress (Figure 5). However, there was no noticeable difference in these parameters under the control and BR-treated plants except APX in a shoot, which significantly increased with BRs (Figure 5C). In particular, Co stress increased the aforementioned attributes by 79.2/133.1%, 302.2/198.5%, 159.6/88.5%, 85.6/117.5%, 160.6/88.3%, and 160.1/90.47% in shoot/root, respectively, as compared to the control plants. Under short-term exposure to moderate HM concentrations, these defense mechanisms can sustain plant growth. However, prolonged or high-concentration exposure overwhelms these protective systems, leading to ROS-induced cytotoxicity, severe damage, and potentially plant death [61].

Under severe oxidative stress conditions, plants require augmented antioxidant support to maintain growth and prevent systemic collapse. This is achieved primarily through enhanced biosynthesis of endogenous antioxidants. In this study, we observed that BRs further increased antioxidants such as CAT (41.2/59.6%), APX (29.4/27.7%), POD (23.7/18.74%), SOD (27.8/21.6%), GSH (23.9/18.74%), and GR (23.9/17.46%) in shoot/root, respectively, as compared to Co stress alone (Figure 5). The enhanced antioxidant activity reduced oxidative stress, as indicated by lower ROS levels and lipid peroxidation. Specifically, BR treatment reduced H_2_O_2_ by 17.79 and 16.66%, O_2_^•−^ by 28.5 and 21.48%, and MDA by 37.5 and 37.9% in shoot and root, respectively, as compared to Co treatment alone (Figure 4). These findings were further validated by fluorescence microscopy which confirmed lower ROS accumulation and improved cellular integrity in BR-treated plants (Figure 4G). This reduction is attributed to the enhanced activation of enzymatic and non-enzymatic antioxidant defense systems, improved ROS scavenging capacity, and the protective role of BRs in stabilizing cellular membranes and mitigating oxidative damage under Co stress. Vardhini and Anjum [62] stated that BR-mediated regulation of the antioxidant defense system plays a vital role in shaping protective responses to mitigate oxidative damage and preserve cellular integrity during abiotic stress. Previously, Wu et al. [37] and Soares et al. [33] demonstrated that BRs enhance cellular antioxidant defenses by activating enzymatic and non-enzymatic components, thereby mitigating ROS-induced oxidative damage under Cr and Pb stress conditions, respectively. Collectively, our findings suggest that BRs confer Co stress tolerance by enhancing the antioxidant defense system, which supports better plant growth and physiological function under Co toxicity.

### 2.6. Effect of BRs on Plant Ultrastructure

**Figure 6:**
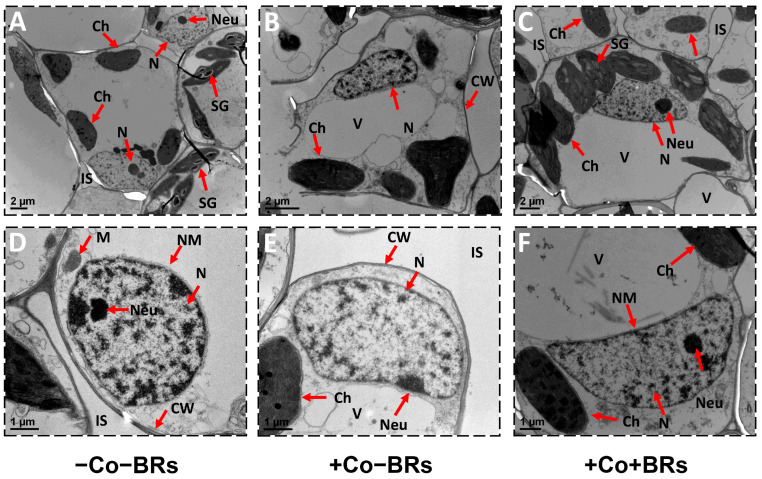
Ultrastructural observations of maize leaf cells under different treatments. Transmission electron micrographs showing (**A**–**C**) overview of cellular ultrastructure and (**D**–**F**) nucleus morphology under (**A**,**D**) control conditions, (**B**,**E**) Co stress, and (**C**,**F**) Co + BR treatment. Co stress induced severe cellular damage, including disorganized membranes, distorted chloroplasts, and nuclear deformation. BR application preserved organelle integrity, maintaining cellular architecture and nuclear morphology under Co stress, indicating a protective role in mitigating ultrastructural damage. Abbreviations: IS, intercellular spaces; M, mitochondrion; V, vacuole; CW, cell wall; Ch, chloroplast; N, nucleus; Neu, nucleolus; NM, nuclear membrane; SG, starch granule.

TEM analysis demonstrated that Co exposure induced significant ultrastructural modifications in mesophyll membrane-bound organelles (Figure 5B,E). The plants subjected to non-stress conditions exhibited intact cellular structures characterized by transparent cell walls, visible cell organelles, a well-developed nucleus featuring a visible nucleolus and a transparent nuclear membrane, as well as developed chloroplasts possessing arranged thylakoids and distinct chloroplast membranes (Figure 6). However, under alone Co stress conditions, the nucleus and chloroplasts in the mesophyll cells exhibited abnormal shapes. The nuclear membrane ruptured and the nucleolus disappeared. The chloroplasts undergo deformation from their typical elongated shape to a more rounded form, and disorganization of grana stacks. Mitochondria also showed abnormal elongation (Figure 3B,E). Previously, ultrastructural damage under HM stress has been widely reported, including Cu [63], Cr [64], Ni [65], and As stress [66]. Ali et al. [59] reported pronounced cellular damage, including disintegrated cell walls, underdeveloped plastoglobules, chloroplasts with swollen thylakoid membranes, and ruptured nuclear envelopes under Co stress. Under adverse conditions, chloroplasts overproduce ROS due to excess photon absorption in PSII and electron leakage to oxygen via PSI. These ROS play dual roles in stress signaling and oxidative damage. PSII generates ^1^O_2_ through energy transfer, while electron leakage and incomplete water oxidation produce O_2_^•−^ and H_2_O_2_, which are further converted to •OH via non-heme iron catalysis [67]. Co generates ROS via Fenton-like reactions, and excess Co ions disrupt the electron transport chain, leading to electron leakage and O_2_^•−^. These reactive species like •OH and O_2_^•−^ cause severe damage to cellular organelles, including the chloroplast and nucleus [18]. In contrast, BR application under Co stress partially alleviated these structural damages, helping to maintain the elongated morphology of chloroplasts and restore nucleolus structure towards normal, compared to the plants treated with Co alone (Figure 6C,F). The reduced oxidative damage can be attributed to the regulatory role of BRs in enhancing antioxidant defense, stabilizing membrane structures, and preserving organelle integrity under stress conditions. Earlier studies reported that exogenous BRs alleviated autotoxicity-induced damage to chloroplasts and thylakoids in cucumber leaves, thereby sustaining photosynthetic efficiency and cellular homeostasis [68]. Basit et al. [69] reported that BR priming positively influenced rice cultivars by contributing to the maintenance of chloroplast ultrastructure under Cr stress. Zhang et al. [70] demonstrated that cellular damage is closely linked to ROS overaccumulation and oxidative stress. Exogenous application of BRs attenuated intracellular ROS levels, safeguarding cellular ultrastructure and preserving cellular integrity. Tang et al. [34] reported severe ultrastructural damage in plants, particularly in chloroplasts, under combined Hg and drought stress. However, the application of BRs mitigated these damages and alleviated the extent of ultrastructural disruption. These findings support our results, highlighting a conserved protective role of BRs in preserving organelle integrity under Co stress through ROS scavenging and membrane stabilization. However, further research is needed to elucidate the precise molecular mechanisms and signaling cascades involved in BR-mediated ultrastructural protection under Co toxicity.

## 3. Materials and Methods

### 3.1. Plant Materials and Growth Conditions

The *Zea mays* L. (Yunrui 668) utilized in this study was procured from the Yunnan Academy of Agricultural Sciences, Kunming, China. Initially, the seeds were subjected to surface sterilization in 5% sodium hypochlorite (10 min) and 70% ethyl alcohol (2 min), followed by rinsing with distilled water. Afterward, the seeds were blotted with a paper towel, arranged on moistened filter paper within a Petri dish, and placed in an incubation chamber under dark conditions at 26 °C for three days before exposure to light. Subsequently, seedlings were transferred to half-strength nutritional media for the initial two days, and then to full-strength [71]. The Hoagland nutritional solution was modified as described in our previous publication [63]. The nutritional solution was continuously aerated with an aeration pump and was changed every three days to prevent depletion. The plants were cultivated in a growth chamber under controlled conditions of 70% relative humidity and a 14/10 h photoperiod cycle.

### 3.2. Experimental Setup and Treatment Induction

The experimental groups were established as follows: (i) control without Co and BR supplementation (−Co − BRs), (ii) exogenously applied BRs (−Co + BRs), (iii) exogenously applied Co (−BRs + Co), (iv) exogenously applied BRs and Co (+BRs + Co). 24-Epibrassinolide served as the source of brassinosteroids, whereas cobalt chloride (CoCl_2_) was used as the cobalt source. 24-Epibrassinolide and CoCl_2_ were procured from Aladdin Bio-Chem Technology Co., Ltd. (Shanghai, China). Treatments were induced on the 12th day of germination for nine days. The concentrations of Co (300 µM) and brassinosteroids (0.1 µM) used in this study were selected based on findings from our earlier experiments, where these doses consistently induced measurable stress responses and demonstrated effective mitigation, respectively [18,72]. The treatments were applied by injecting the desired concentration into the nutrient media, which was refreshed every three days. After nine days of treatment, the plants were harvested, and the following parameters were measured.

### 3.3. Plant Growth and Photosynthetic Parameters

The plants were harvested, separated into roots and shoots, and their fresh weight and length were measured. The measured sample was washed and stored in a drying oven at 65 °C to determine its dry weight. The remaining samples were stored at −80 °C for further measurements. For chlorophyll contents, fresh leaf segments were fragmented and stored in 85% acetone for six hours, and the aliquots were analyzed using a spectrophotometer following the procedure outlined by Khan et al. [73]. The gas exchange parameters, including net photosynthetic rate (*Pn*), transpiration rate (*Tr*), and stomatal conductance (*gs*), were assessed following 7 days of stress and hormonal treatments prior to the experiment’s harvest. A portable photosynthetic system reader (Li-Cor 6400XT, Lincoln, NE, USA) was employed for this purpose, with measurements obtained at the apex of the fully expanded leaf [74]. The measurement conditions remained constant, with a maximum airflow rate of 700 µmol s^−2^. The photosynthetically active radiation (PAR) was measured at 1600 µmol m^−2^, while the carbon content was kept at 360 µmol m^−1^.

### 3.4. Determination of Cobalt Contents

For Co contents, the samples were washed in ethanol to desorb any loosely attached Co ions from the surface and put in a drying oven. The dried root and shoot samples were crushed into a fine powder using a mechanical grinder. For metal analysis, 0.1 g of the powdered samples was incubated in concentrated nitric acid (HNO_3_) for 1 h, followed by digestion at 120 °C and then at 140 °C for 2 h using a Dry Thermo Unit (Taitec, Tokyo, Japan). After digestion, the samples were diluted to the desired volume with deionized water. The concentrations of Co in the leaf and root tissues were quantified using inductively coupled plasma mass spectroscopy (ICP-MS) [75].

### 3.5. Measurements of Endogenous Phytohormones

A Plant Epibrassinolide (EBR) ELISA Kit (Jingmei Biotechnology, Yancheng, China) was used to measure endogenous EBR content in plant tissues, following the manufacturer’s instructions. Briefly, fresh leaf tissue (0.1 g) was homogenized in pre-cooled PBS buffer (0.01 M, pH 7.4) at a 1:9 (*w*/*v*) ratio to prepare a 10% homogenate. The homogenate underwent centrifugation at 8000× *g* (30 min) at 4 °C, and the supernatant was subsequently collected. BR concentration was then quantified by measuring absorbance at 450 nm using a microplate reader (multifunctional, BioTek, San Diego, CA, USA), following the ELISA kit protocol. The levels of ABA, IAA, GA_3_, and JA were analyzed according to the method described by Pan et al. [76]. Fresh maize leaf tissue (0.2 g) was extracted and purified. Internal standards (d5-IAA, d6-ABA, and d2-GA_3_) were obtained from Sigma (St. Louis, MO, USA) and added as follows: 25 μL of d6-ABA (0.25 ng/μL), 2 μL of d5-IAA (2 ng/μL), and 50 μL of d2-GA_3_ (2 ng/μL). Then, 0.5 mL of extraction buffer (isopropanol/ H_2_O/concentrated HCl, 2:1:0.002, *v*/*v*/*v*) was added, and the mixture was shaken at 100 rpm for 30 min at 4 °C. Subsequently, 1 mL of dichloromethane was added and shaken for 30 min. After centrifugation, the lower phase was collected and concentrated using a nitrogen evaporator. The dried residue was dissolved in 100 μL of aqueous methanol (H_2_O, 1:1, *v*/*v*) and centrifuged at 1200× *g* for 5 min. The supernatant was then transferred to an HPLC (Agilent Technologies 1100, Cerritos, CA, USA) vial for analysis with a flow rate of 0.8 mL/min.

### 3.6. Polyphenols, Soluble Sugar, and Total Protein

Fresh leaf samples (0.5 g) were finely ground into a powder, and 80% ethanol was used to extract total phenolics and flavonoids. Extraction continued until the pellet became colorless, followed by centrifugation at 4000× *g* for 10 min. The resulting supernatants were stored for analysis. The Folin–Ciocalteu method with sodium carbonate was used to determine the total phenolic content, and absorbance was measured at 650 nm. Total flavonoid content was quantified by measuring the absorbance of the extracts at 430 nm. To determine soluble sugar content, 0.5 g of fresh seedlings was taken, homogenized in 10 mL of distilled water, and centrifuged at 8000× *g* for 15 min at 4 °C. Then, 1 mL of the supernatant was mixed with 4 mL of anthrone reagent and water bathed for 5 min at 100 °C. After cooling to room temperature for at least 5 min, absorbance was measured at 620 nm. Sugar content was quantified using a standard calibration curve. For soluble protein estimation, 0.1 g of fresh seedlings was ground in 1 mL of phosphate-buffered saline (PBS, pH 7.4) and centrifuged at 8000× *g* (10 min) at 4 °C, and the supernatant was collected. An aliquot (5 μL) was mixed with 250 μL of Coomassie Brilliant Blue G-250 reagent and the absorbance was read after 30 min. Absorbance was measured at 595 nm, and protein content was calculated using a standard curve based on known protein concentrations.

### 3.7. Hydrogen Peroxide (H_2_O_2_) and Superoxide Anion (O_2_^•−^) Quantification and Imaging

Hydrogen peroxide (H_2_O_2_) levels were determined using a commercial H_2_O_2_ detection kit (Cat. No. BC3590, Solarbio, Beijing, China) following the manufacturer’s protocol. Briefly, 0.1 g of fresh leaf and root tissues were ground into a fine powder under chilled conditions using a multi-sample tissue homogenizer (Tissuelyser-48, Xian Yima Optoelec Co., Ltd., Xi’an, China). The homogenate was mixed with 1 mL of extraction reagent and centrifuged at 8000× *g* at 4 °C (10 min). The supernatant was collected, and the detection reagents were added as per the kit instructions. Absorbance was measured at 415 nm using a spectrophotometer, and H_2_O_2_ concentration was calculated based on the standard curve provided in the kit. Superoxide anion (O_2_^•−^) content was assessed using a superoxide detection kit (Cat. No. BC1295, Solarbio, China). Ground tissue samples were suspended in 1 mL of buffer solution and centrifuged at 12,000× *g* for 10 min. The pellet was washed three times with buffer and centrifuged at 10,000× *g* for 10 min after each wash. Subsequently, 20 µL of buffer was added to the pellet and centrifuged again at 10,000× *g* for 5 min. The final absorbance was recorded at 530 nm using a microplate reader, and O_2_^•−^ levels were quantified as instructed by the manufacturer.

ROS were visualized in situ using a laser confocal scanning microscope (Olympus FV3000, Tokyo, Japan) with fluorescent probes for H_2_O_2_ and O_2_^•−^ detection [63]. Fresh leaf and root segments were rinsed with phosphate-buffered salt (PBS, 50 mM, pH 7.8), followed by incubating in 20 µM 2′,7′-dichlorodihydrofluorescein diacetate (H_2_DCFDA) for 30 min in the dark to detect H_2_O_2_. For O_2_^•−^ detection, the samples were incubated in dihydroethidium (DHE) for 60 min. After staining, samples were fixed on glass slides with coverslips and observed using excitation/emission wavelengths of 488 nm/500–600 nm for DCF and 518 nm/500–600 nm for DHE. Three biological replicates were analyzed per treatment, with two technical replicates per sample.

### 3.8. Lipid Peroxidation Assay and Antioxidant Enzymatic Activities

Lipid peroxidation was estimated by quantifying MDA content, following the protocol described by Zhou and Leul [77]. Briefly, 0.3 g of fresh plant tissue was homogenized into a fine powder using a multi-sample tissue grinder under chilled conditions. The homogenate was extracted in 6 mL of phosphate-buffered saline (PBS; 50 mM, pH 7.8) and centrifuged at 12,000× *g* for 15 min at 4 °C. The resulting supernatant was mixed with 5% trichloroacetic acid (TCA) and 5% thiobarbituric acid (TBA), then incubated in a water bath at 95 °C for 10 min and subsequently cooled down on ice. After cooling, samples were centrifuged at 4800× *g* for 10 min, and the absorbance was recorded at 532 nm and 600 nm (UV-2600, Shimadzu, Japan). MDA content was determined by subtracting the non-specific absorbance measured at 600 nm from the specific absorbance at 532 nm. POD activity was determined by monitoring the oxidation of guaiacol. The reaction mixture consisted of PBS (50 mM, pH 7.8), 300 mM H_2_O_2_, and 1.5% guaiacol, which was combined with the enzyme extract. The increase in absorbance was recorded at 470 nm for 1 min using a spectrophotometer. SOD activity was assayed following the method of Zhang et al. [78], based on the inhibition of nitroblue tetrazolium (NBT) reduction. The reaction system contained 75 μM of NBT, 20 μM of riboflavin, 100 μM of EDTA-Na2, and 130 mM of methionine, mixed with the enzyme extract. Absorbance was measured at 560 nm after illuminating the reaction mixture. CAT activity was determined by measuring the decomposition rate of H_2_O_2_. The reaction mixture included PBS (50 mM, pH 7.8) and 300 mM H_2_O_2_, to which the enzyme extract was added. The reduction in absorbance was monitored at 240 nm for 30 s. APX activity was quantified using the method of Nakano and Asada [79]. The reaction mixture consisted of PBS (50 mM, pH 7.8), 300 mM H_2_O_2_, and 7.5 mM ascorbic acid, combined with the enzyme extract. The decline in absorbance was measured at 290 nm for 1 min using a UV spectrophotometer.

### 3.9. Determination of Reduced Glutathione and Glutathione Reductase Contents

For the determination of GSH, 0.5 g of fresh tissue was pulverized in a mortar with 2 mL of 2% metaphosphoric acid and subsequently centrifuged at 5500× *g* for 20 min according to Griffith [80]. From the resulting supernatant, 100 μL was added to a reaction mixture containing 700 μL of NADPH (0.3 mM), 100 μL of DTNB (6 mM), and 100 μL of distilled water. After a few minutes, 10 μL of GR was added, and the absorbance was measured at 412 nm. GR activity was assessed using the protein extract. The decrease in absorbance at 340 nm was monitored to track the oxidation of NADPH in a reaction mixture, as described by Foyer et al. [81]. Enzyme activity was quantified in units per milligram of protein.

### 3.10. Plant Ultrastructure Analysis

Ultrastructural observations were made via transmission electron microscope (TEM) according to Azhar et al. [82]. Leaf disks excluding the midrib were excised and fixed overnight in 2.5% glutaraldehyde prepared in 100 mM phosphate buffer (pH 7.0). Post-fixation was carried out using 1% osmium tetroxide (OsO_4_) for 2 h at room temperature, followed by three successive washes with the same phosphate buffer (100 mM, pH 7.0) for 15 min each and then dehydrated through a graded ethanol series (30%, 50%, 70%, 80%, 90%, 95%, and 100%) for 15 min at each step. Samples were then treated with absolute acetone twice, each for 20 min. The dehydrated samples were infiltrated with a resin–acetone mixture in a 1:1 ratio for 1 h and a 1:3 ratio for 3 h and then embedded in pure resin and polymerized overnight at room temperature. Ultrathin sections (~70 nm) were prepared using an ultramicrotome (Leica EM UC7, Burladingen, Germany), and sections were subsequently stained with uranyl acetate and alkaline lead citrate. The stained sections were mounted on copper grids and examined under a TEM (Hitachi H-7650, Japan) to evaluate cellular and subcellular structures.

### 3.11. Statistical Analysis

Experiments were conducted with three biological replicates. Statistical analysis was performed using one-way analysis of variance (ANOVA) in Statistix software (version 8.1). Results are presented as mean ± standard deviation (SD). Differences among means were assessed via Fisher’s LSD test at *p* < 0.05. Treatments with different lowercase letters indicate significant differences. The graphical presentation was created with GraphPad Prism 8.0.

## 4. Conclusions

We hypothesized that BRs, as plant growth-promoting hormones, may enhance tolerance to Co stress by modulating physiological processes and maintaining hormonal homeostasis. This hypothesis was grounded in our previous findings highlighting the protective role of BRs against HM stress [72]. Our results showed that exogenous BR supplementation significantly improved plant growth and biomass by elevating phenolic and flavonoid levels, as well as improving soluble sugar and total protein contents. BRs also improved antioxidant enzymes (SOD, POD, CAT, APX, GSH, GR) and reduced oxidative damage by reducing ROS and MDA accumulation, thereby preserving cellular ultrastructure. Additionally, BRs increased the endogenous levels of BR and other growth hormones (IAA, JA, GA) in the shoot and inhibited Co uptake in the root and its translocation to the shoot. These physiological and biochemical improvements translated into enhanced plant growth, biomass production, photosynthetic efficiency, and gas exchange parameters. Collectively, the findings underscore the potential of BRs as effective modulators of plant stress responses, offering a promising strategy to enhance crop resilience under HM-induced toxicity. Nonetheless, further investigations are needed to elucidate the molecular mechanisms involved and to optimize BR application for broader stress tolerance and sustainable agricultural practices. Additionally, research should also investigate the crosstalk of BRs with other growth modulators (nanoparticles, biochar, etc.) to identify potential synergistic interactions that could fine-tune their individual efficacy and collectively enhance plant growth and stress tolerance.

## Data Availability

Data will be made available on request.
